# “What Was His Name, Again?”: A New Method for Reducing Memory-Based Errors in an Adult False-Belief Task

**DOI:** 10.5964/ejop.v16i2.1998

**Published:** 2020-05-29

**Authors:** Marea S. Colombo, Charlotte Bremer, Julien Gross, Jamin Halberstadt, Harlene Hayne

**Affiliations:** a Department of Psychology, University of Otago, Dunedin, New Zealand; Kingston University, London, United Kingdom

**Keywords:** Theory of Mind, false belief, adults, training

## Abstract

Despite considerable interest in the development of Theory of Mind (ToM) during early childhood, until recently, there has been little consideration about whether and how ToM skills continue to change into adulthood. Furthermore, the false-belief task, which is believed to capture the underlying mechanisms of ToM, is rarely used in studies of ToM with adults; those tasks that do assess false-belief understanding may be confounded by incidental task demands, such as complex narratives and excessive memory requirements, making it difficult to isolate adults’ true ToM skills, much less to compare them with the skills of children. Here, we adapted a task developed by Valle, Massaro, Castelli, and Marchetti (2015, https://doi.org/10.5964/ejop.v11i1.829) to assess false-belief understanding in adults. Participants were randomly assigned to one of three conditions. In the reading condition, participants read a story about the unexpected transfer of a ball between three brothers. In the video condition, participants watched a video version of the same story. Finally, in the training condition, participants were first trained on the names of the characters, before watching the video. Although condition did not affect participants’ ability to correctly answer a standard false belief question (“Where does X think Y thinks the ball is?”), participants in the training condition used more mental state language to justify their responses (“Why does X think Y thinks the ball is here?”), and this improved performance was mediated by improved memory for the story details. We conclude that at least some “failures” of ToM use may be due to an inability to understand, recall, or communicate complex information in a ToM task, raising important questions about how best to measure ToM in adults (and children) in the future.

Theory of Mind (ToM) refers to our ability to predict, explain, and interpret others’ beliefs, desires, intentions, and behavior (Wellman, 1990; Wellman, Cross, & Watson, 2001). Researchers have now shown that ToM is related to a number of individual differences in the way we interact with others, including both psychological skills like social understanding, language interpretation, and empathy, as well as social outcomes like number of close friends (Astington & Jenkins, 1999; Ibanez et al., 2013; Peterson, Slaughter, Moore, & Wellman, 2016; Stiller & Dunbar, 2007).

To date, most research on ToM has focused on preschool- and early-school-age children, assessing their abilities via some version of a “false-belief” task (for reviews, see Wellman et al., 2001; Wellman & Liu, 2004). For example, in the classic task developed by Wimmer and Perner (1983), children listen to a story in which “Maxi” puts some chocolate in cupboard X. Maxi then leaves the room and while he is gone, his mother moves the chocolate from cupboard X to cupboard Y. After hearing the story, participants are asked where Maxi will look for the chocolate (a so-called “first-order” false-belief question). To answer correctly (i.e., that Maxi will look in cupboard X), participants must differentiate between what they know to be the true location of the chocolate, and Maxi’s knowledge of where he left the chocolate. In the conceptually-similar “unexpected contents” task (Perner, Leekam, & Wimmer, 1987), participants are presented with a crayon box and asked what they believe is inside the box. Unsurprisingly, children typically say ‘crayons,’ but the experimenter reveals the contents of the box to actually be candy. Participants are then asked about what Mary, someone who has never seen the true contents of the container, will think is in the box: crayons or candy. Once again, to answer correctly, children must differentiate between what they know to be true and what Mary believes.

A meta-analysis of data collected using first-order false-belief tasks has shown that between the ages of 3 and 6 years, children make substantial progress in their understanding of false belief (Wellman et al., 2001). Wimmer and Perner (1983) found that although the majority of 4- to 5-year-olds could not answer first-order false-belief questions, 91% of 6- to 7-year-olds and 100% of 8- to 9-year-olds answered at least one correctly, independent of question framing (e.g., whether the experimenter asks where the protagonist will look, or what the protagonist believes or knows; Wellman et al., 2001). Over this same period, children develop some understanding that people can have desires (Repacholi & Gopnik, 1997; Wellman & Woolley, 1990) and knowledge (Pratt & Bryant, 1990) that differ from their own; they also begin to understand that sometimes people show one emotion, but feel another (Harris, Donnelly, Guz, & Pitt-Watson, 1986).

While there has been considerable research on the emergence of ToM in young children, far less research has focused on the subsequent development of ToM in older children and adults. Clearly, the simple false belief tasks that are commonly used with young children are developmentally inappropriate for use with older participants and researchers have now turned to new tasks to help them understand age-related changes in ToM beyond early childhood. In this line of work, researchers have focused on a range of components of ToM including cognitive, visual, and affective ToM (understanding what people think, see and feel respectively; Imuta, Henry, Slaughter, Selcuk, & Ruffman, 2016). For example, Symeonidou, Dumontheil, Chow, and Breheny (2016) used a “Director Task” to assess the development of visual ToM in children (aged 9-13-years old), adolescents (aged 14-18-years old), and adults (aged 19-29-years old). Participants were asked to follow the instructions of a Director, who had a limited view of a set of objects arrayed on shelves, all of which were in full view of the participant. Participants were explicitly told to only move objects that the director would be able to see. In the control condition, there was no director and the participants were told to ignore the objects in slots with a gray background. Children and adolescents made more errors than did adults in the director condition, but not in the control condition, providing evidence that the use of ToM continues to improve with age beyond early childhood (see also, Dumontheil, Apperly, & Blakemore, 2010; Keysar, Lin, & Barr, 2003).

The Strange Stories Task is another task that has been specifically designed for use with adults. In this task, participants are asked to explain deceptive behaviours (white lies, jokes, sarcasm, etc.), which requires taking a character’s cognitive and affective perspective into account. Maylor, Moulson, Muncer, and Taylor (2002) used an adapted version of the Strange Stories Task (Happé, 1994) to assess the developmental decline of ToM in the course of normal aging. They tested young, young-old, and old-old participants (mean ages: 19, 67 and 81 years respectively) and found that participants’ performance on this task declined as a function of age; 81-year-olds performed worse than 67-year-olds who performed worse that 19-year-olds. Furthermore, in their second experiment, Maylor et al. also assessed whether participants’ ToM performance relied on their need to remember specific information in the task. Despite eliminating the need to recall the details of the story, older adults performed worse on the ToM task. These results are consistent with those obtained using emotion recognition tasks and suggest that ToM may peak in early adulthood, and decline with age independent of decline in other cognitive functions like memory (Sullivan & Ruffman, 2004).

Although research conducted with the Strange Stories Task suggests that ToM ability might peak during early adulthood, research using other measures suggests that young-adult ToM is potentially less reliable than it appears. For example, in another task, “Reading the Mind in the Eyes,” participants are asked to select the label that best describes the emotional or mental state of a character (e.g., angry, reflective) based on their eyes alone (Baron-Cohen, Wheelwright, Hill, Raste, & Plumb, 2001). Rutherford, Baron-Cohen, and Wheelwright (2002) subsequently developed an auditory version of Baron-Cohen et al.’s (2001) task in which participants are asked to identify a speaker’s state based on his or her brief statements. In both versions, normal adults between the ages of 20 and 48 years performed below ceiling, failing to consistently identify another’s emotional or mental state. Subsequent researchers have identified a number of individual difference factors that are related to performance on the Eyes Task including sex (Carroll & Chiew, 2006), interest in fiction novels (Mar, Oatley, & Peterson, 2009), and level of prosocial orientation (Declerck & Bogaert, 2008).

Although research with adults has now enhanced our understanding of ToM (Apperly, Back, Samson, & France, 2008; Carroll & Chiew, 2006), this work has rarely used the false-belief task that is typically used with children, an omission that not only prevents our ability to chart ToM from early childhood across the lifespan, but also risks missing the essence of the construct: the distinction between the contents of one’s own and others’ minds (Bradford, Jentzsch, & Gomez, 2015). Of the few exceptions, Duval, Piolino, Bejanin, Eustache, and Desgranges (2011) assessed young (*M*_age_ = 23.80 years), middle-aged (*M*_age_ = 52.55 years), and older adults (*M*_age_ = 70.14 years) on a battery of ToM tasks. As part of this ToM battery, participants completed a standard false belief task in which participants read a series of 3-panel comic strips that depicted characters who had different knowledge about a given event. For example, one story charted an interaction between friends, Marie and Francois. In the first panel, Marie tells Francois that she wants to get her hair cut and then they can meet at a café for lunch. In the second panel, Marie changes her mind and curls her hair instead. In the final panel, Francois arrives and sees two females facing the bar, one with long curly hair and one with short hair. Participants are asked to decide whether Francois will approach the female on the left (with long curly hair) or on the right (with short hair). Given that Francois does not know that Marie has changed her mind, participants would be credited with using ToM if they said that Francois would approach the female on the right. Consistent with other research, results from this study indicated that older participants showed deficits on a false-belief task, compared to young and middle-aged adults.

In another study of this kind, Valle, Massaro, Castelli, and Marchetti (2015) used a false-belief task modeled on the classic tasks that have been used with preschool- and early school-age children (Wimmer & Perner, 1983). Valle et al. asked participants to read a story about the unexpected transfer of a ball between three brothers. After reading the story, participants answered a series of false belief questions. Although first-order false-belief questions (Where will X look for the ball?) are typically used with children, Valle et al. assessed both second-order (Where does X think Y will look for the ball?) and third-order (Where does X think Y thinks Z will look for the ball?) false-belief questions. Valle et al. found that participants correctly answered more second-order false-belief questions than third-order false belief questions. On the third-order false belief questions, in particular, adolescents performed significantly worse than did young adults, but even young adults performed well below ceiling, suggesting that higher-order false-belief understanding might have a long developmental trajectory.

A potential limitation of Valle et al.’s (2015) study, however, is the semantic complexity of the materials, an issue endemic to the false-belief task, even those used with children. In Valle et al., the story was presented to participants in writing, only once, and involved multiple transitions between multiple characters (e.g., “*While Mark is in the kitchen, James takes the ball to play, puts it in the closet, and leaves the room to go to the bathroom. At this point, Luke takes the ball and plays with it. Luke hits the ball with his foot awkwardly, and the ball drops behind a large wardrobe.*”). These procedural details raise the possibility that some participants may have failed the task not because they lacked ToM, but because they could not remember specific details of the story.

Another important issue in Valle et al.’s (2015) study is that, consistent with prior research on the false-belief task used with children, the researchers defined ToM in terms of participants’ answers to the false-belief questions alone (e.g., “where does X think Y will look for the ball?”). Although ToM is defined as the ability to attribute mental states to other people (Premack & Woodruff, 1978), the procedure typically used in false-belief tasks does not require the participant to articulate the reason for their choice. Although it might be the case that a correct answer is based on the participant’s understanding of another person’s thoughts, it might also be based on another form of reasoning. Additionally, an incorrect answer does not necessarily preclude ToM reasoning, but rather may reflect confusion, forgetting, or a whole host of other non-measured factors.

In the present study, we continued to explore ToM in adults using the task originally developed by Valle et al. (2015), but we made two important changes to the task. First, to overcome some of the memory demands of the task, we developed a video version of the materials that was closely based on Valle et al.’s written version. The video version provided a more realistic and dynamic stimulus. Manipulations similar to those used in the current experiment have been shown to improve performance on other ToM tasks such as the eyes task (Back, Jordan, & Thomas, 2009). We predicted that this stimulus, particularly when it was paired with additional training on the characters’ names would lead to greater memory, enhancing ToM performance (see also Back et al., 2009). Second, to gain a better understanding of adults’ ToM reasoning, we not only asked them to answer standard ToM questions, but we also asked them to justify their answers.

## Method

### Participants

Ninety-three 18- to 34-year-old undergraduate university students (42 female, *M*_age_ = 20.20 years, *SD* = 2.37) were recruited through a university participant database and satisfied a small portion of course assessment by completing a worksheet based on the experiment. (Three participants were excluded from the final sample, one because he had dyslexia, and two due to experimenter error and additional participants were recruited to ensure each group had 30 participants.) The research was reviewed and approved by the University of Otago Human Ethics Committee, which is accredited by the national Health Research Council and whose guidelines are consistent with those of the American Psychological Association.

### Materials

The false-belief task was modelled on the task used previously by Valle et al. (2015) in their study with adolescents and adults. Participants were randomly assigned to one of three experimental conditions (reading, video, or training; *n* = 30 participants in each condition). Participants in the *reading* condition were given the following story to read at their own pace:

Mark, James and Luke are three brothers. Mark, James and Luke are in their bedroom. Mark is playing with a ball, James is playing on the computer and Luke is reading a book on the bed. After the game, Mark decides to go to the kitchen to eat a snack and puts the ball in a closed box. James and Luke see Mark put the ball in the box. While Mark is in the kitchen, James takes the ball to play, puts it in the closet, and leaves the room to go to the bathroom. At this point, Luke takes the ball and plays with it. Luke hits the ball with his foot awkwardly, and the ball drops behind a large wardrobe. Luke is unable to recover the ball, so he climbs onto the bed and continues reading. James meets Mark in the hallway and tells him that he put the ball in the closet. After several minutes, the two brothers enter in the bedroom. James goes to the computer, and Luke makes a very clear gesture to tell Mark that the ball is behind the wardrobe. James does not see this gesture. Mark wants to play again with the ball.

For participants in the video condition, instead of reading the story, they watched a videotaped dramatization of the story twice. Professional actors performed the role of each character; participants were introduced to each character at the beginning of the video and each character wore a different coloured t-shirt to make them easier to differentiate.

The procedure for the *training* condition was identical to the video condition, except that, prior to watching the video, the experimenter showed the participant a picture of each character, said the character’s name, and tested participants’ memory for the names by covering the names, pointing to the characters in turn and asking “What is his name?” Once participants could correctly identify all of the characters’ names without any errors, they watched the video twice.

In summary, our reading condition is a replication of Valle et al. and our experimental conditions include both a video and a training condition. Based on our estimates of how long it took to read the story, participants in the video and training conditions watched the video twice.

### Primary Dependent Measures

Immediately after they finished reading or viewing the story, participants were asked to respond and justify their answers^i^ to one second-order false-belief question and two third-order false-belief questions: (1) Where does Luke think that James will look for the ball? Why? (2) Where does Luke think that James thinks that Mark will look for the ball? Why? (3) Where does James think that Luke thinks that Mark will look for the ball? Why? Participants verbally responded to the questions and their responses were recorded on a digital voice recorder. Questions were repeated and clarified if requested, but the experimenter did not remind the participant of the story content.

Next, participants’ memory for the scene was assessed with nine questions, always in the same order, in which they were asked to identify the characters (e.g., “Who was playing with the ball first?”), where each character put the ball, and who saw him place the ball there (see Appendix).

### Additional Measures

Participants’ expressive language and word retrieval ability were tested using the Expressive Vocabulary Test (EVT; Williams, 2007) and receptive language ability was tested using the Peabody Picture Vocabulary Test (PPVT; Dunn & Dunn, 2007). To control for variations in age between participants, all raw scores on the EVT and PPVT were converted to age-standardized scores.

### Procedure

Participants were tested individually in the laboratory. On arrival, they were seated at a table opposite a female experimenter who outlined the purpose and procedure of the study, and obtained written informed consent. Participants completed the language assessments first, followed by the false-belief task. One female experimenter conducted all of the false-belief tasks, and for some participants, a second female experimenter administered the language tests. At the end of the experimental session, participants were debriefed.

## Results

### Demographic and Language Variables

As shown in Table 1. there were no significant differences between the experimental conditions in terms of participants’ age, *F*(2, 87) < 1, *p* = .461, η^2^ = .018; sex, χ^2^(2) = 3.03, *p* = .220; PPVT score, *F*(2, 87) < 1, *p* = .610, η^2^ = .011; or EVT score, *F*(2, 87) = < 1, *p* = .458, η^2^ = .018. Scores on the EVT ranged from 73 to 133, and on the PPVT from 76 to 126. There were 2 participants who scored more than 1 standard deviation below the standardized mean (100) on both the PPVT and the EVT. Three additional participants scored more than 1 standard deviation below the standardized mean (100) on the EVT. The results did not differ when these outliers were excluded; thus all participants were included in the analyses. As expected, scores on the EVT and PPVT were positively correlated, *r*(88) = .77, *p* < .010.

**Table 1:** Means and Standard Deviations for Age and Language Test Scores as a Function of Condition

Variable	Condition					
	Reading (*N* = 30, 18 Female)		Video (*N* = 30, 24 Female)		Training (*N* = 30, 22 Female)	
	*M*	*SD*	*M*	*SD*	*M*	*SD*
Age	20.47	2.86	19.93	1.76	20.63	2.03
PPVT^a^	107.23	10.39	105.07	10.79	104.87	9.35
EVT^a^	106.20	12.99	102.57	13.20	102.73	11.93

*Note*. PPTV = Peabody Picture Vocabulary Test; EVT = Expressive Vocabulary Test.

^a^Standardized score.

### Memory

Two research assistants, unaware of participants’ condition assignment, coded participants’ responses to the nine memory questions. Participants were assigned a score of 1 if they correctly answered a memory question and 0 if they provided an incorrect response to a memory question. Coders showed perfect (100%) agreement.

**Figure 1:**
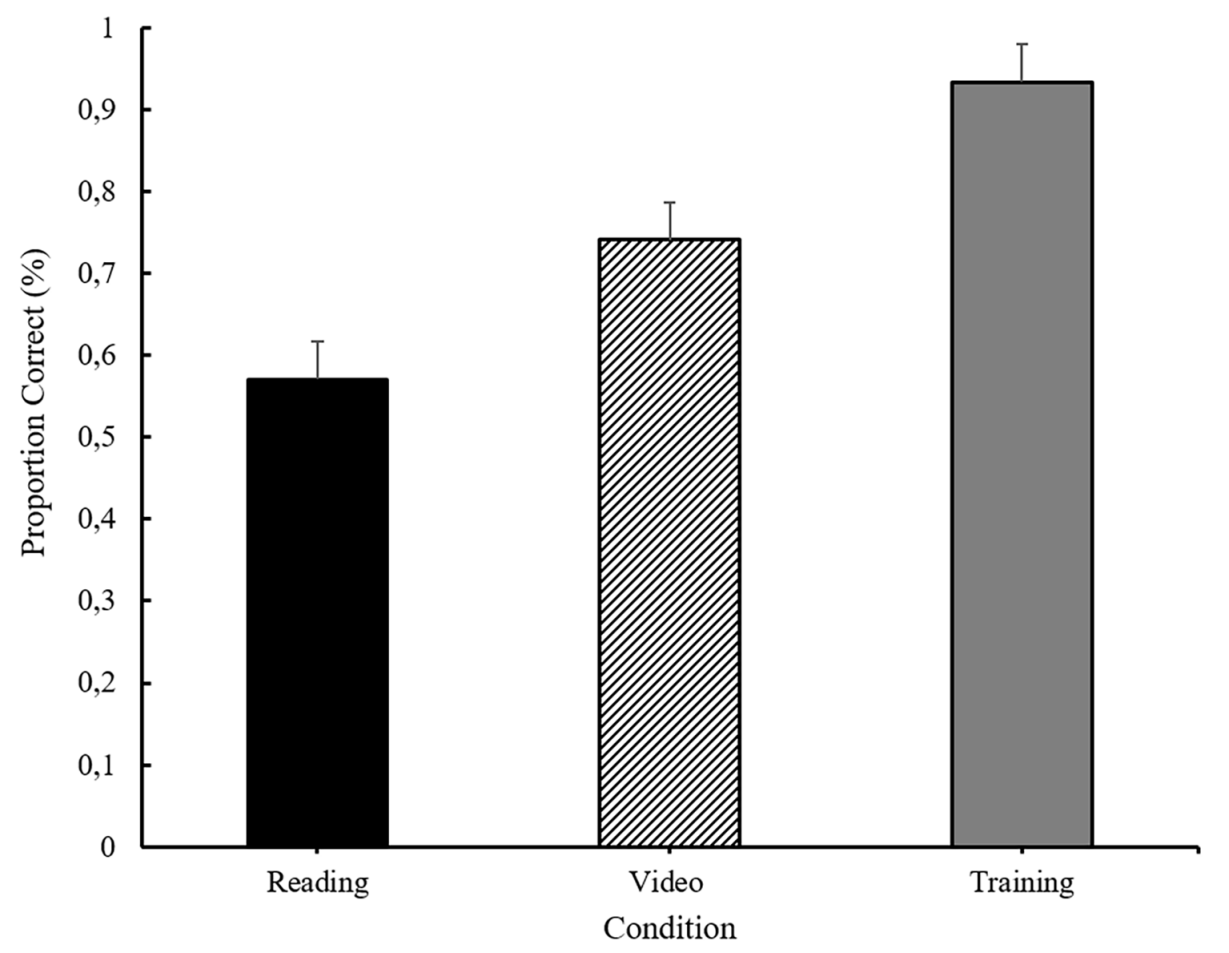
The mean proportion of memory questions answered correctly as a function of experimental condition. Error bars reflect standard errors of the means.

The memory scores are shown in Figure 1. These scores were analyzed using a univariate ANOVA, which revealed a significant main effect of condition, *F*(2, 87) = 15.59, *p* < .001, η^2^ = .26. Independent sample *t*-tests revealed that participants in the training condition exhibited significantly better memory for the story than those in the video condition, *t*(58) = 3.62, *p* < .010, or the reading condition, *t*(58) = 5.61, *p* < .001, which were also significantly different from each other, *t*(58) = 2.26, *p* = .028. Memory scores did not correlate with scores on the PPVT or the EVT either within each condition (PPVT; reading: *r*(28) = .250, *p* = .183, video: *r*(30) = .10, *p* = .605, training: *r*(28) = -.21, *p* = .260; EVT reading: *r*(28) = .29, *p* = .120, video: *r*(28) = -.06, *p* = .756, training: *r*(28) = -.04, *p* = .823) or across the entire sample (PPVT: *r*(88) = .04, *p* = .687; EVT: *r*(88) = .03, *p* = .806).

### False-Belief Task Accuracy

Two research assistants, unaware of participants’ experimental condition, coded participants’ responses to the false-belief questions (e.g., “Where does Luke think James will look for the ball?”). Participants were assigned a score of 1 if they identified the correct location of the ball, and a score of 0 if they identified the incorrect location of the ball. Coders again showed perfect agreement.

Recall that participants were asked one second-order false-belief question and two third-order false-belief questions. According to a McNemar’s exact test, there was no difference in participants’ accuracy on the two Level-3 questions, *p* = 1.000. Given this result, and to provide a more conservative test of our hypotheses^ii^, participants were coded as correct for Level-3 ToM if they correctly answered *either* of the Level-3 questions.

**Figure 2:**
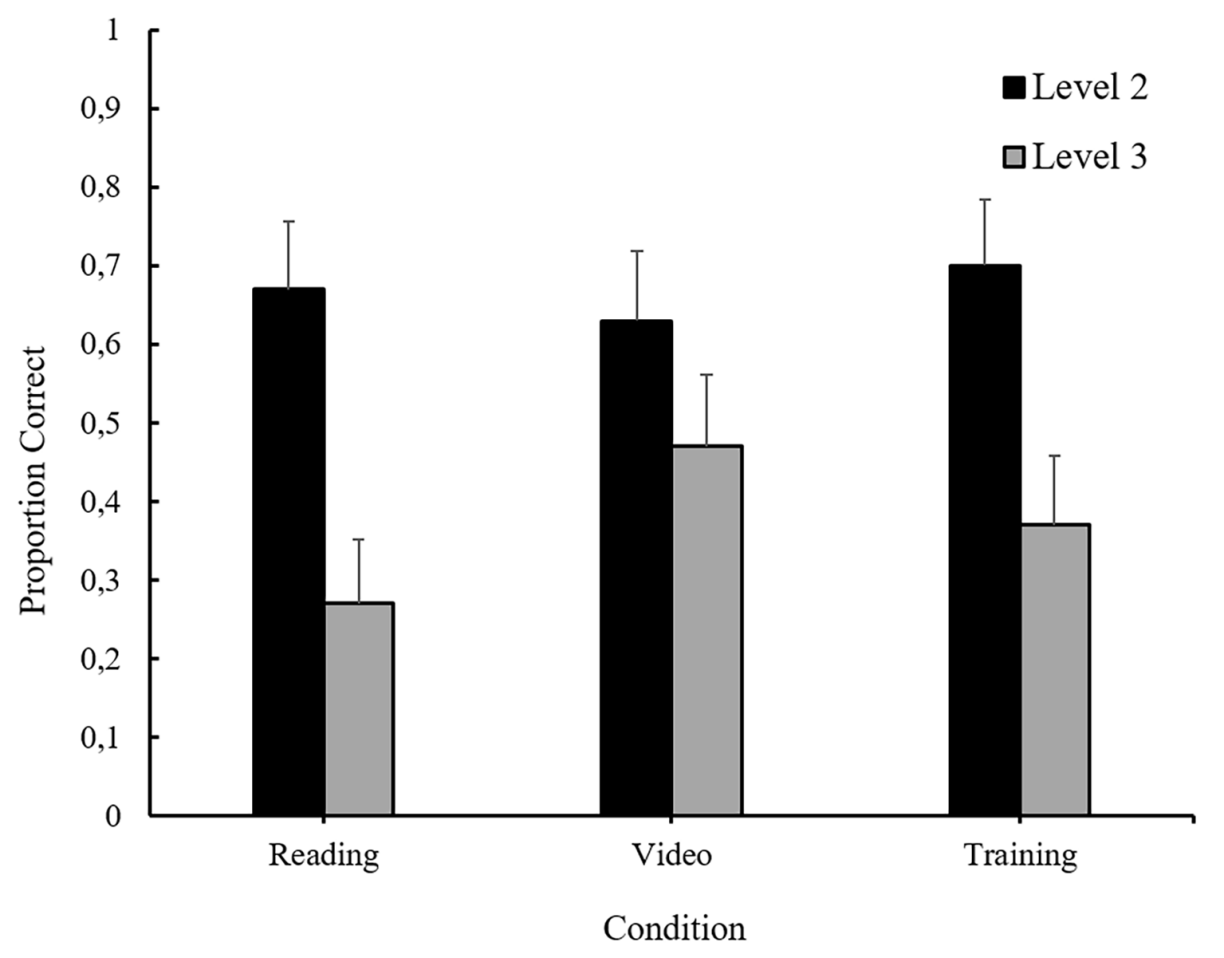
The proportion of participants who correctly answered the false-belief questions as a function of experimental condition and question level. Error bars reflect standard errors of the means.

Participants’ performance on the false-belief test is shown in Figure 2. Their performance was analyzed as a function of condition and question level, using a generalized estimating equations (GEE) model commonly used to analyze repeated-measures binary data (Liang & Zeger, 1986). The analysis revealed a significant main effect of question level, such that more participants correctly answered the Level-2 question, *M* = 0.67, 95% CI [0.56, 0.76], than the Level-3 questions, *M* = 0.36, 95% CI [0.27, 0.47], χ^2^(1) = 17.11, *p* < .001. There was neither an effect of condition, nor an interaction.

### False Belief Task Justifications

Two research assistants, unaware of participants’ condition assignment, coded participants’ justifications for their use of mental-state reasoning, independent of the accuracy of their response. Participants were assigned a score of 1 if the justification included a description of the characters’ mental states (e.g., “he did not know that the ball had moved”), and 0 otherwise (e.g., “he put the ball there”). Coders showed acceptable interrater reliability (Kappa = .81), and all disagreements were resolved through discussion.

**Figure 3:**
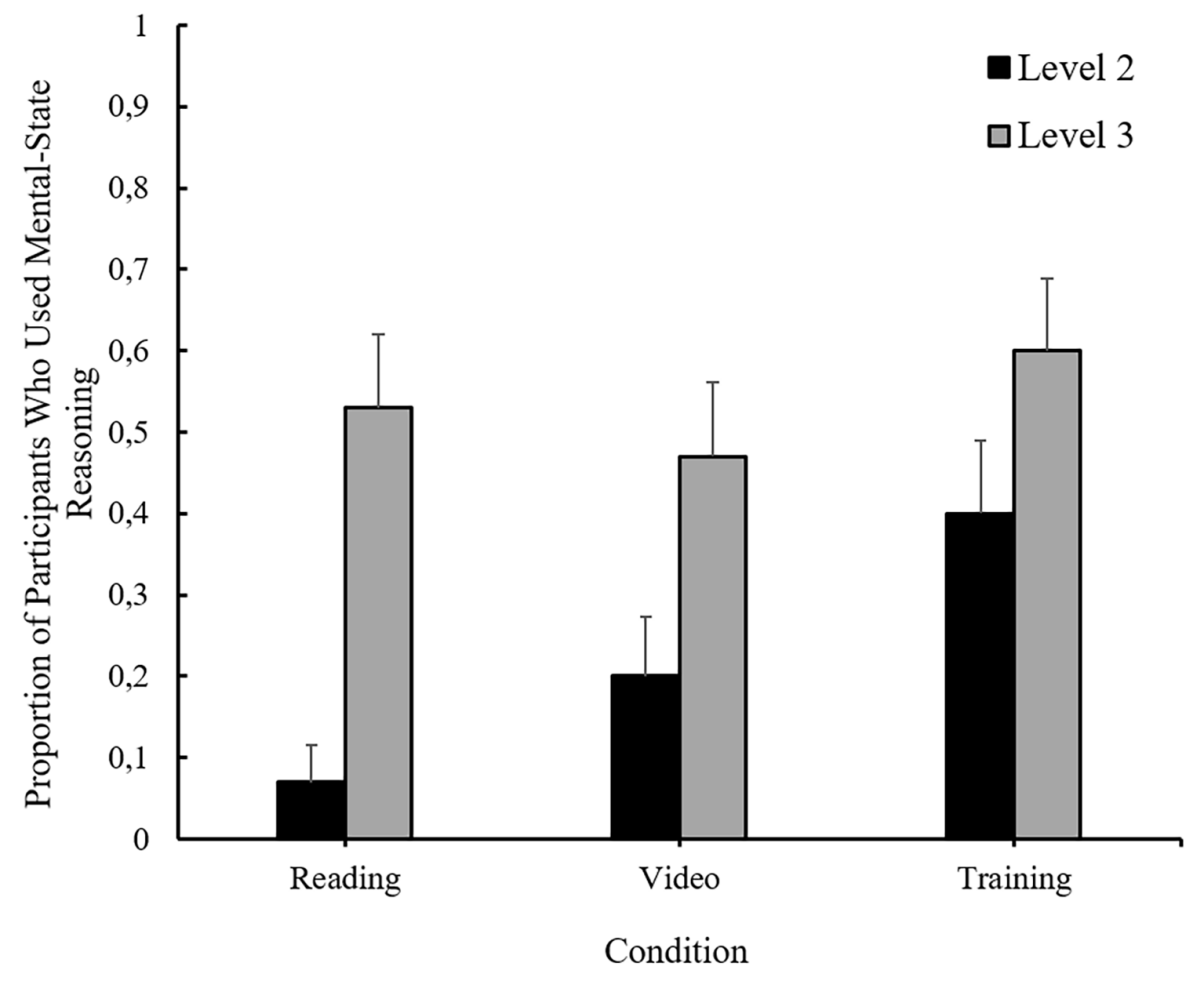
The proportion of participants who used mental-state reasoning in their justification as a function of experimental condition and question level. Error bars reflect standard errors of the means.

As with the accuracy data, justifications were analyzed with a 3 (Condition) x 2 (Question Level) GEE model, treating the Level-3 ToM measure as binary (i.e., participants were credited with ToM use if they used mental state language in response to either Level-3 question) and the data are shown in Figure 3. There was a significant main effect of question level; more participants used mental-state justification on the Level-3 questions, *M* = 0.53, 95% CI [0.43, 0.63], than on the Level-2 question, *M* = 0.19, 95% CI [0.11, 0.30], χ^2^(1) = 28.16, *p* < .001. There was also a significant effect of condition; compared to participants in the reading condition, *M* = 0.22, 95% CI [0.11, 0.41], more participants in the training condition, *M* = 0.50, 95% CI [0.35, 0.65], used mental-state justification in their responses, χ^2^(2) = 7.40, *p* = .007. Additionally, more participants in the video condition used mental-state justification than in the reading condition, however this difference did not reach significance, χ^2^(2) = 2.11, *p* = .15. The Condition X Question Level interaction was also marginally significant, χ^2^(2) = 5.78, *p* = .056. As shown in Figure 3 there were no differences between participants’ performance on the Level-3 questions as a function of condition, χ^2^(1) = 1.07, *p* = .590. For Level-2 questions, however, participants in the training condition, *M* = 0.40, 95% CI [0.24, 0.58], were increasingly more likely to reference character’s mental states to justify their responses than were participants in the video condition, *M* = 0.20, 95% CI [0.09, 0.38] *SE* = 0.073, and in the reading condition, *M* = 0.07, 95% CI [0.02, 0.23], χ^2^(1) = 9.77, *p* = .008.

**Figure 4:**
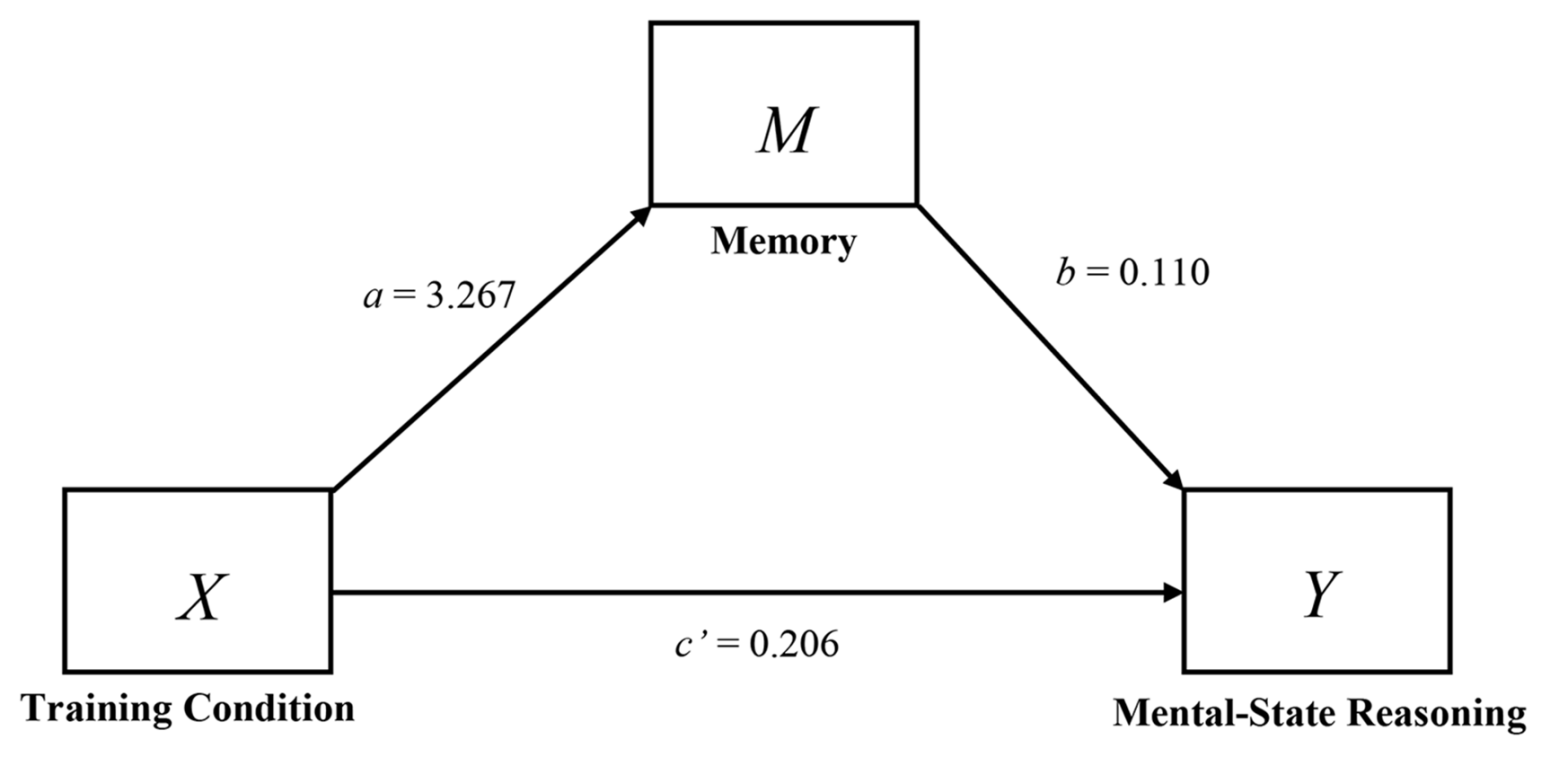
Model of the effects of the training condition mediated by memory.

Given that participants in the training condition performed better on both the memory task and the justification task, we examined whether the improved justification scores were mediated by improved memory in the training condition. A test of mediation using Hayes’ (2012) Process procedure (Model 4, 10,000 bootstrap resamples) revealed that the bias-corrected confidence interval for the indirect effect (0.360) did not include zero (0.088 to 0.643)^iii^. Our findings support a mediation hypothesis as the training condition did not affect mental-state use independent of memory (c’ = 0.206, *p* = .50), suggesting that our new method reduced unnecessary task demands that may have previously masked ToM ability. The model is illustrated in Figure 4 and Table 2.

**Table 2:** Model Coefficients for the Mediating Effects of Memory on Theory of Mind Use

Antecedent	Consequent					
	2. M^a^			3. Y^b^		
	Coefficient	*SE*	*p*	Coefficient	*SE*	*p*
1. X	3.267^c^	0.586	< .01	0.206^e^	0.302	.497
2. M	-	-	-	0.110^d^	0.048	.023
3. Y				-	-	-
4. Constant	5.133	0.414	< .01	0.134	0.305	.662

*Note*. X = Training condition; M = Memory; Y = Mental-state reasoning.

^a^*R*^2^ = .26; *F*(2, 87) = 15.585; *p* < .01. ^b^*R*^2^ = .11; *F*(2, 87) = 3.532; *p* < .018. ^c^See effect *a* in Figure 4. ^d^See effect *b* in Figure 4. ^e^See effect *c’* in Figure 4.

### Other Analyses

Although scores on the EVT did not correlate with the total number of correct answers, or scores on the memory task, they did correlate with the number of mental-state justifications used, *r*(90) = .24, *p* = .023. Given that the EVT was associated with number of mental-state justifications, this variable could have accounted for some of the variance in performance. A test of mediation using Hayes’ (2012) Process procedure (Model 4, 10,000 bootstrap resamples) revealed that the bias-corrected confidence interval for the indirect effect (-0.038) included zero (-0.150 to 0.024), suggesting that EVT score cannot account for the variance in performance. The length of time that participants took to read the story was not correlated with their false-belief accuracy or their justification responses. There was also no correlation between accuracy on the false-belief task and use of mental-state reasoning in the justification questions, *r*(90) = .16, *p* = .123.

## Discussion

Despite considerable interest in the development of ToM during early childhood, until recently, there has been little consideration about whether and how ToM skills might continue to change into adulthood. Although the false-belief task is believed to capture the underlying mechanisms of ToM, most tests of ToM in adults have focused on other aspects of this skill such as emotion recognition (Baron-Cohen et al., 2001) and visual perspective taking (Keysar et al., 2003). Those tasks that do assess false-belief understanding (e.g., Valle et al., 2015) may be compromised or confounded by incidental task demands, such as complex narratives and memory requirements, making it difficult to isolate adults’ true ToM skills, much less to compare them with the skills of children. In addition, when testing children or adults, relying solely on answers to the false-belief questions alone may obscure critical differences in ToM understanding.

One goal of the current experiment was to assess whether a visual false-belief task, combined with initial training on the identities of the characters in the false-belief task, would improve adults’ ToM performance. A second goal was to examine adults’ rationale for the answers they provided. To achieve these goals, we evaluated three modes of presentation on adults’ memory for story details, the accuracy of their false-belief understanding, and their use of mental-state reasoning to justify their answers. Participants had better memory for the story when they saw a video presentation and were trained on the characters’ names than did participants who saw the same video but without training, or simply read the story, suggesting that cognitive load may be a critical limitation in the standard administration of the false-belief task for adults, and presumably children as well. Indeed, participants in the reading condition recalled scarcely more than half of the information about who did what and what they were supposed to be reasoning. Conversely, participants in the video condition showed better memory, and in the training condition, almost perfect memory, for the characters and their behavior, suggesting that the use of these enhanced formats could improve performance on the false-belief task and, more importantly, provide a more accurate estimate of adults’ ToM abilities.

Surprisingly, although participants in the training condition did show improved accuracy on memory questions, they were no more accurate on the false-belief questions than were participants in the reading group. Conversely, participants in the training condition were more likely to use mental-state reasoning to explain their answer, independent of accuracy. Furthermore, memory scores mediated the improved performance on reasoning, suggesting that at least some “failures” of ToM use can be explained by an inability to understand, recall, or communicate the complex information in a ToM task.

Another, perhaps more important implication of our results relates to the disjunction between responses to ToM questions and use of ToM reasoning. ToM tasks in the literature vary in terms of whether they assess ToM use directly, or infer ToM use from “correct” answers to test questions. In the current experiment, we assessed both—participants’ ToM use was inferred from correct answers about the presumed location of the ball, but was also assessed directly via participants’ justifications for their answers (irrespective of whether the answers were correct or not)—and illustrates how an exclusive focus on accuracy in a false-belief paradigm (or any other measure of ToM) could miss important variability in actual competence.

Our results show that accuracy is neither necessary nor sufficient for ToM reasoning: participants can employ ToM concepts even without answering the questions correctly, or answer correctly via means other than ToM reasoning; reliance on accuracy alone can therefore produce under- and over-estimation of individuals’ ToM skills, respectively (see also Robinson & Mitchell, 1995). Almost two thirds of our participants (65%) failed to provide mental-state justification for their answers; similarly, children may answer ToM questions correctly with no understanding of why their choice is correct, or arguably answer questions correctly without invoking ToM at all (cf. Robinson & Mitchell, 1995; Wimmer, Mayringer, & Landerl, 1998). Even if participants fail to reveal ToM reasoning to experimenters for other reasons (e.g., self-presentation, lack of motivation), the gap between their self-reported solutions to ToM questions, and the process by which they arrived at them, shows that the two questions should be treated as independent sources of insight into what other people are thinking.

The present findings raise important questions regarding the presumed “gold standard” of the False Belief Task. In the present experiment, participants’ ability to answer the false-belief questions was not correlated with their use of ToM to justify their response. That is, participants sometimes provided the correct answer without an adequate justification and sometimes they provided the incorrect answer but used mental-state language to support their choice. Other researchers have also assessed the relationship between answers and explanations in the false-belief task and have found mixed results. For example, Wimmer, Mayringer, and Landerl (1998) presented children with two false-belief stories, one in a prediction condition and one in an explanation condition. In the explanation condition, participants were specifically asked, “why would Sally look in Y.” In the prediction condition, on the other hand, participants were first asked the standard false-belief question, “Where will Sally look for her ball?” In this latter condition, participants were corrected if they provided an erroneous response, and then were asked to explain why the protagonist would look in that given location. Although Wimmer and Mayringer found that children performed equally well on both versions of the task, others have reported differences when children are asked to explain a false-belief task as opposed to predicting a protagonist’s actions (e.g., Robinson & Mitchell, 1995). Taken together, these studies, and our own study, raise important questions about the best method of assessment in a standard false-belief task.

Consistent with Valle et al. (2015), the adults in the current experiment were nowhere near ceiling in terms of either accuracy or reasoning, even with the benefit of training. It is possible that participants could and would have performed even better given additional training or motivation (Ickes, 2011), but it is clear that, for whatever reason, they did not exhibit the kind of ToM ability one would expect if ToM were more or less fully developed by age 9 (e.g., Wimmer & Perner, 1983). The importance of motivation becomes even more evident because at least some of the skills associated with ToM, such as suppression, cognitive adjustment, and attribution, theoretically require cognitive effort; they are “controlled” rather than “automatic” processes (Schneider & Shiffrin, 1977). According to Bargh (1994), while automatic processing is unintentional, unconscious, uncontrollable and efficient (Frankish & Evans, 2009), controlled processing is characterized by relatively slow, effortful, and often conscious decision making. Although early theorists argued that a mental process was either automatic or controlled (e.g., Johnson & Hasher, 1987), there is now consensus that most mental processes lie on a continuum and involve elements of both (Bargh, 1989, 1994). Importantly, if ToM does require controlled processes, then it follows that its use requires not only the access to sufficient cognitive resources, but also the motivation to use ToM skills in any given situation. An interesting avenue for future research would be to identify the cognitive and motivational factors that moderate performance in adults, and to get an accurate estimate of individuals’ true abilities across the lifespan. It will also be important to replicate these findings with a range of different stimulus materials and test procedures.

The present data have both theoretical and practical implications. From a theoretical perspective, our results, and those of others, suggest that false-belief understanding continues to develop until at least early adulthood, and even then, there are large individual differences in skill. We contend that one reason that adults (and children) may “fail” false-belief tasks is that the tasks themselves include incidental demands on memory and cognitive processing. Indeed, we found that participants remembered more details of a ToM interaction when some of those incidental demands were removed (i.e., when participants first learned the characters’ names), and that this improved memory mediated the use of mental-state justification (but not accuracy) in the false-belief task. The video and training methods we developed here open new opportunities for ongoing research on ToM in both children and adults that may provide a more complete understanding of how ToM is acquired and used across the life-span.

From a practical perspective, much of the previous research on ToM has focussed on when individuals acquire the use of ToM. On the basis of previous research, adults appear to have a fully mature grasp of ToM and thus they should be able to easily employ their skills. The present research questions the presumed competence of adult ToM and also provides opportunities to understand when, and under what conditions adults will employ their ToM abilities. Research investigating children’s ToM, however, has found that ToM ability is associated with a host of positive social outcomes. It is entirely likely that individual differences in ToM in adults may predict other success factors in a wide range of professions and social situations.

## Appendix

### Memory Questions

1. Who was playing with the ball first?

2. Who was playing on the computer?

3. Who was reading a book on the bed?

4. Where did Mark put the ball?

5. Who saw him put it there?

6. Where did James put the ball?

7. Who saw him put it there?

8. Where did Luke kick the ball

9. Who saw him kick it there?
